# Autoimmune Hepatitis: A Review of Current Diagnosis and Treatment

**DOI:** 10.1155/2011/390916

**Published:** 2011-05-15

**Authors:** Ashima Makol, Kymberly D. Watt, Vaidehi R. Chowdhary

**Affiliations:** ^1^Division of Rheumatology, Department of Medicine, Mayo Clinic College of Medicine, Rochester, MN 55905, USA; ^2^Division of Gastroenterology and Hepatology, Department of Medicine, Mayo Clinic College of Medicine, Rochester, MN 55905, USA

## Abstract

Autoimmune hepatitis (AIH) is a chronic inflammatory disorder characterized by periportal inflammation, elevated immunoglobulins, autoantibodies, and a dramatic response to immunosuppression. An environmental agent is hypothesized to trigger an immune-mediated attack directed against liver antigens in genetically predisposed individuals. A plethora of clinical presentations can be seen ranging from chronic indolent disease to fulminant hepatic failure, and diagnosis requires exclusion of other causes of liver disease. Corticosteroid therapy must be instituted early and modified in an individualized fashion. Treatment decisions are often complicated by the diverse clinical manifestations, uncertainty about natural history, evolving ideas about treatment end points, and a multitude of alternative immunosuppressive agents. Achieving normal liver tests and tissue is the ideal treatment end point, but needs to be weighed against the risk of side effects. Decompensated patients may benefit from early liver transplantation. Long-term prognosis is excellent with early and aggressive initiation of therapy. Our paper discusses AIH, giving a detailed overview of its clinical presentation, risk factors, immunopathogenesis, up-to-date diagnostic criteria, current updates in therapy with a brief discussion of AIH in pregnancy, and long-term implications for cirrhosis and hepatocellular carcinoma in AIH patients.

## 1. Background

Autoimmune hepatitis (AIH) is a chronic inflammatory disease of unknown etiology characterized by the presence of circulating autoantibodies, hypergammaglobulinemia, necroinflammatory changes on hepatic histology, and a dramatic response to immunosuppressive therapy. Earliest descriptions include those by Amberg in 1942 [1] and Leber in 1950 [2] describing a form of chronic liver disease prevalent among young women and characterized by an excessive increase in serum protein and gamma-globulins. In 1951, Kunkel et al. termed the condition “hypergammaglobulinemic chronic hepatitis” [3]. Since then, it has been known by various names including chronic active hepatitis, chronic aggressive hepatitis, plasma cell hepatitis, and autoimmune chronic active hepatitis. Cowling and Mackay coined the term “lupoid hepatitis” after they noted the association of this entity with autoimmune syndromes and the LE cell phenomenon [4]. 

The disease is rare with a mean incidence of 1-2 per 100,000 and a point prevalence of 11–17 per 100,000 [5, 6]. Although more frequently seen in young women (sex ratio 3.6 : 1), it can affect children and adults of all ages and ethnicities [7, 8]. A minority of patients may present with acute liver failure and need liver transplantation, but for the majority, the prognosis of AIH is good and mostly determined by response to corticosteroid therapy. In general, long-term survival and average life expectancy are excellent and estimated to be comparable to the normal population [9].

## 2. Classification

The classification of AIH into different types is based on serum autoantibody profiles. Type I AIH is characterized by the presence of antinuclear antibody (ANA), anti-smooth muscle antibody (SMA), or both and constitutes 80% of AIH cases. About 25% have cirrhosis at presentation, and association with other autoimmune diseases is common (celiac disease, ulcerative colitis, autoimmune thyroid disease) [10, 11]. Type 2 AIH is characterized by the presence of anti-liver kidney microsomal (LKM) 1 and/or anti-LKM3 and/or anti-liver cytosol 1 (LC1) [12, 13] antibodies. Most patients are children, acute severe presentation can occur, and progression to cirrhosis commonly ensues [14]. 

In patients who are negative for conventional antibodies and AIH is strongly suspected, additional tests can be done including perinuclear antineutrophil cytoplasmic antibodies (pANCA), actin (anti-actin), soluble liver antigen (anti-SLA), asialoglycoprotein receptor (anti-ASGPR), chromatin, and liver cytosol type 1 (anti-LC1). In our experience, 10–15% patients do not have either ANA, SMA, or anti-LKM1 at presentation, but 25% of these will have detectable conventional antibodies later in their course. Another 10–20% of the seronegative patients at presentation will have pANCA or anti-SLA. Overall, approximately 5% will have no currently available markers long term.

### 2.1. Etiopathogenesis 

Although the exact etiopathogenesis is unknown, AIH, like many autoimmune diseases, is thought to be caused by environmental triggers and failure of immune tolerance mechanisms in a genetically susceptible host. These triggers may be of viral or drug etiology, but most cases have an unknown trigger. Triggers may share epitopes that resemble self-antigens, and molecular mimicry between foreign antigens and self-antigens is the most frequently proposed initiating mechanism in type 2 AIH where the autoantigen is known. Repeated exposures to the triggering antigen, in turn, may trigger autoreactive organ-specific responses.

### 2.2. Genetic Associations

AIH is a complex polygenic disease and different populations may have different genetic and environmental triggers and genetic association varies in study populations. The human leukocyte antigen (HLA) genes on chromosome 6 are the most commonly described association with AIH. HLA associations vary by ethnicity and have been summarized in Table 1. HLA may be associated with age at presentation, disease severity, and response to therapy. How the HLA genes predispose to disease is not exactly known but is likely due to their role in autoreactive T cell selection and autoantigenic peptide presentation. Different susceptibility alleles like HLA DR*β*1*0301, DR*β*1*0401, DR*β*1*0404, and DR*β*1*0405 share a “common motif,” namely, amino acids LLEQKR or LLEQRR at position 67-72 of class II HLA, whereas the resistant alleles DR*β*1∗1501 encodes ILEQAR [20, 21]. In contrast, HLA-DR*β*1*1501, encodes for the ILEQAR motif [21] and is associated with protection from AIH. Substitution of a lysine or arginine to alanine at position 71 is postulated to change the polarity and charge of the peptide binding groove of the major histocompatibility complex thereby influencing autoantigenic peptide presentation. However, these associations are not absolute and significant geographic differences exist, for example, in Japan DR2 (DRB1*1501) is a weak susceptibility rather than a resistance allele [22] and in South American children DRB1*1301 is a strong susceptibility allele [23]. Furthermore, patient numbers in many HLA studies are low.

A form of AIH can be seen in 20% of patients with autoimmune polyendocrinopathy-candidiasis-ectodermal dystrophy (APECED) syndrome. APECED is a monogenic, autosomal recessive disorder characterized by hypoparathyroidism, adrenal insufficiency, and chronic mucocutaneous candidiasis. APECED is caused by mutations in a transcription factor relevant to immune tolerance called AIRE (autoimmune regulator) on chromosome 21q223. AIRE is expressed in medullary epithelial and dendritic cells within the thymus and regulates clonal deletion of autoreactive T cells. The liver autoantigens associated with APECED are cytochrome P450 (CYP) 1A2, CYP2A6, and CYP2D6 [24–26]. This is the only syndrome involving AIH that exhibits a Mendelian pattern of inheritance, and genetic testing and counseling for the patient and family members are warranted [24].

### 2.3. Immunopathogenesis

Full insight into the pathogenesis of AIH remains elusive. The liver is part of the lymphoid system with the normal lymphocyte population mainly residing in the portal tracts. AIH is an inflammatory disorder of the liver involving multiple components of the immune system including T cells, B cells, and cytokines. Hepatocytes isolated from AIH patients are coated with immunoglobulins and are susceptible to antibody-dependent cellular cytotoxicity (ADCC) when exposed to autologous mononuclear cells bearing F_c_ receptors [40]. CYP2D6, an important cytoplasmic enzyme is targeted by anti-LKM1 antibodies and plays a crucial role in liver damage. Mice immunized with plasmid containing human CYP2D6 antigenic region and human formiminotransferase cyclodeaminase (another autoantigen), have established a murine model for autoimmune hepatitis type 2 [41]. These mice develop autoantibodies, elevation in transaminases, along with portal and periportal inflammatory infiltrate. Another model using adenovirus vector containing human CYP2D6 infection of CYP2D6 transgenic mice had focal hepatocyte necrosis and hepatic fibrosis [42]. These models will aid the development of more therapeutic options in the management of autoimmune hepatitis.

Studies have demonstrated presence of cytotoxic cells in both T and non-T cell compartment of peripheral blood from AIH patients. This cytotoxic activity is higher in patients with active disease but seen in only 40% of patients in remission [43]. Patients with AIH have a ten fold higher frequency of liver-specific T cells compared to normal subjects [32]. In patients with predisposing HLA allele DR*β*1*0701, CD4 T cells are able to recognize autoantigen CYP2D6 and secrete interferon-*γ* [44]. In addition CD8+ T cells have been isolated from portal tract infiltrate. CD8 T cells have cytotoxic capability, are capable of secreting IFN-*γ*, and their responses correlate with disease activity [45].

Defects in numbers and function of regulatory cells (T regs) have been demonstrated in AIH [46]. T regulatory cells normally control or limit immune responses by acting as immunoregulators, preventing the proliferation and effector function of autoreactive T cells. In patients with AIH, T-regs are defective both in number and function. The number of T regs is decreased more so at disease presentation than at drug-induced remission. Their level correlates inversely with levels of anti-SLA and anti-LKM-1 autoantibody titers [46]. The T reg numbers and function improve during remission but are never normal. Longhi et al. in their study demonstrated that Tregs generated under CYP2D6-specific conditions and cocultured with semimature dendritic cells are highly effective at controlling autoreactive T cells, thus providing a potential tool for immunotherapy in type 2 AIH [47]. T regs may, therefore, be an attractive therapeutic target, but more studies are needed to elucidate this better.

### 2.4. Environmental Factors

Several drugs have also been implicated as triggers for AIH including Infliximab [48], Minocycline [49], Atorvastatin [50], diclofenac, isoniazid, *α*-methyldopa, nitrofurantoin, and propylthiouracil and Hepatitis A vaccine [51]. Herbal agents such as black cohosh and dai-saiko-to have been proposed to induced AIH [52]. The exact reason for drug-induced AIH is not known but may be due to hepatotoxic effect of these chemicals, upregulation of proteins expression (P450s, immunoregulatory proteins), or related to the drug acting as a hapten by modifying the hepatic protein, making them immunogenic. Drug-induced AIH may improve after discontinuation of offending agent, thus initial observation is warranted.

Viruses such as hepatitis A, B, or C, in addition to measles have been implicated as triggers for AIH. ANA and SMA can occur in diverse causes of acute and chronic hepatitis including alcoholic, nonalcoholic fatty liver disease, and viral hepatitis. They are usually low titer, background reactivities that should not alter diagnosis or management. If clinical concern for autoimmune hepatitis exists, antiactin antibodies can be checked as they increase the specificity of SMA testing for diagnosing AIH [53]. Anti-LKM1 has been found in as many as 10% of patients with chronic hepatitis C and is different from the anti-LKM1 found in classic autoimmune hepatitis [54]. Molecular mimicry at the B-cell level between a structural motif of CYP2D6 and HCV proteins could explain the production of anti-LKM1 antibodies in HCV-infected patients [55].

## 3. Clinical Manifestations

Women constitute at least 70% of cases, and 50% are younger than 40 years; however, age at onset may range from infancy to the elderly. AIH has diverse presentations with 25–34% of patients presenting with asymptomatic liver test abnormalities. Forty percent of patients may present with an acute onset, but the presentation of severe fulminant hepatic failure is rare [56]. Children or elderly more commonly present with cirrhosis. Presenting symptoms may include fatigue, lethargy, malaise, arthralgia of small joints, anorexia, nausea, abdominal pain, and dark urine. These symptoms are nonspecific and contribute to the delay in diagnosis. Asymptomatic patients commonly become symptomatic, and thus need to be monitored. Clinical manifestations may vary by ethnicity; the presentation is acute and icteric in Alaskan [57] native patients, cholestatic in Aboriginal North American, African, Asian, and Arab patients, mild in Japanese patients [58], but severe and rapidly progressive in Somali patients [59]. Cirrhosis occurs in as many as 85% of black North American patients [60]. Differences in etiologic agents and genetic factors may account for this variation. 

Physical examination may be normal, but may also reveal hepatomegaly, splenomegaly, jaundice, and stigmata of chronic liver disease. Findings such as acne, hirsutism, obesity, and amenorrhea in young women are rarely seen. Other autoimmune diseases such as Hashimoto thyroiditis, type 1 diabetes, rheumatoid arthritis, systemic lupus erythematosus, ulcerative colitis/Crohn's disease, and celiac disease can be seen in 20% of patients [61]. 

Chung et al. described a novel subtype of AIH characterized by infiltration of IgG4-positive plasma cells [62]. This subtype is associated with higher serum levels of IgG, AIH severity scores, and, more importantly, an excellent response to prednisone therapy for induction and maintenance of remission. Positive IgG4 staining is suggested by the authors as a surrogate marker for the subtype of AIH that may respond well to corticosteroid therapy alone. Whether this is a form of classic AIH or a distinct entity awaits a more extensive description of its clinical and immunohistological features.

## 4. Diagnosis of Autoimmune Hepatitis

The diagnosis of AIH requires the presence of characteristic clinical features and exclusion of other chronic liver conditions, such as viral hepatitis, drug-induced hepatitis, fatty liver disease, alcohol related liver disease, Wilson's disease, alpha 1 antitrypsin deficiency, or hemochromatosis.

### 4.1. Laboratory Features

Laboratory studies typically show elevation of aspartate aminotransferase (AST) and alanine aminotransferase (ALT) levels, but levels are generally <500 U/L, but on rare occasions can range between 500–1000 U/L. Some patients may have high conjugated bilirubin and alkaline phosphatase necessitating exclusion of extrahepatic biliary obstruction, cholestatic forms of viral hepatitis, drug-induced disease, primary biliary cirrhosis (PBC), and primary sclerosing cholangitis (PSC). The alkaline phosphatase rarely exceeds 4 X normal and generally remains <2 times normal. Another characteristic laboratory feature of AIH is hypergammaglobulinemia, with a selective increase in IgG, which is 1.2–3.0 times higher than the upper level of normal [63]. It should be noted that HLA typing has not been endorsed as a diagnostic or prognostic tool.

### 4.2. Autoantibodies

The characteristic circulating autoantibodies seen in AIH include ANA, SMA, and (LKM-1) autoantibodies. A list of the important autoantibodies and their autoantigenic targets is summarized in Table 2 [27]. They are helpful in diagnosis as well as for classification of AIH into type 1 and type 2 diseases. The reader is referred to excellent reviews for description of methodology, sensitivity, and assay performance [27, 39, 64]. Except for pANCA, which is readily available and can be positive in 50–90% of type I AIH, only antiactin can be easily in measured in some laboratories. Recently, antibodies to cyclic citrullinated peptides (CCP) have been described in 9–11% of patients with AIH in absence of rheumatoid arthritis. These patients have a propensity to develop cirrhosis and liver failure [65]. Antimitochondrial antibodies are sometimes present in patients with AIH and an overlap syndrome of AIH and PBC should be considered in these patients [66].

### 4.3. Diagnostic Scoring System

A diagnostic system was proposed by the International Autoimmune Hepatitis Group (IAIHG) in 1993 and subsequently updated in 1999 [66, 67]. In 2008, they proposed a simplified set of diagnostic criteria to facilitate early recognition and initiation of adequate immunosuppressive treatment [68]. These included the presence of specific autoantibodies (ANA, SMA, LKM antibody, SLA antibody) in moderate to high titers, hypergammaglobulinemia, typical histological pattern on liver biopsy, and exclusion of viral hepatitis. These criteria have a lower sensitivity (85% versus 100%) but higher specificity (99% versus 93%) than the original criteria and are good at identifying patients with all typical characteristics of a classic case of AIH [69, 70]. However, Miyake et al. showed that 30% of males, 23% of patients with acute clinical presentation, and 46% patients negative for ANA were not diagnosed with AIH by simplified criteria even though they met the original criteria [69]. Therefore, it fails to adequately identify cases with atypical features which is an important point to keep in mind.

### 4.4. Histological Diagnosis

**Figure 1:**
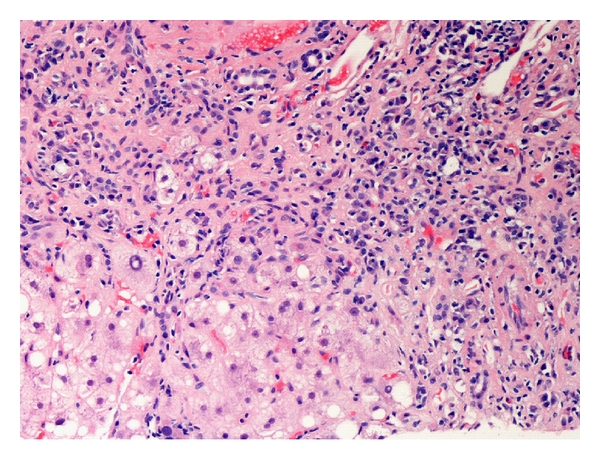
Interface hepatitis demonstrated by infiltration of lymphoplasmacytic infiltrate into the hepatic parenchyma typical of autoimmune hepatitis.

The histologic hallmark of AIH is a lymphoplasmacytic periportal infiltrate invading the limiting plate, also called piecemeal necrosis or “interface hepatitis” (Figure 1) that eventually progresses to lobular hepatitis. There is often an abundance of plasma cells and eosinophils are frequently present. The portal lesion typically spares the biliary tree. A lobular, or panacinar hepatitis is also frequently observed. Fibrosis is present in all but the mildest forms of AIH. It causes distortion of the hepatic lobule and the appearance of regenerative nodules, resulting in cirrhosis [71]. Many patients with acute presentation may have chronic features on liver biopsy indicating a subclinical phase of disease and several patients with mild clinical disease may have advanced fibrosis on biopsy. Of important note is the fact that the fibrosis and even cirrhosis in AIH is reversible to a significant degree with immunosuppressive therapy unlike in other chronic liver diseases.

### 4.5. Radiology

There are no specific imaging techniques to confirm the diagnosis of autoimmune hepatitis. In adults with both AIH and IBD, cholangiographic changes suggestive of PSC are present in up to 44% patients and may affect therapy and prognosis [72]. In children with AIH, autoimmune sclerosing cholangitis can be present with or without inflammatory bowel disease [73].

## 5. Therapy

### 5.1. Indications for Treatment

AIH is a treatable chronic liver disease in the majority of the cases. Untreated patients with active histologic inflammation have worse overall survival. Histologic presence of bridging or multilobular necrosis is associated with progression to cirrhosis in 82% cases and a 5-year mortality of 45% in untreated patients [74]. In asymptomatic patients with inactive cirrhosis (defined as no or limited inflammation), corticosteroid therapy has not shown to improve survival. Patients without cirrhosis who undergo treatment have a 10–20 year survival probability more than 80%, similar to the general population [71]. Retrospective analysis of patients with mild disease has demonstrated the possibility of long-term survival without treatment, but very careful follow-up is required. Untreated patients may, rarely, recover spontaneously, but improvement is less common than treated patients, and long-term survival is lower [75]. AIH can have unpredictable and varying disease activity and ultimately the majority of patients with active inflammation will warrant therapy. Indications for treatment are listed in Table 3 and are based on the presence and severity of hepatic inflammation. The indications are also reflective of risk factors for disease progression as severely abnormal liver enzyme elevation, incapacitating symptoms, histological presence of interface hepatitis, bridging necrosis, or multiacinar collapse portend a worse prognosis without treatment.

### 5.2. Treatment Regimens

**Figure 2:**
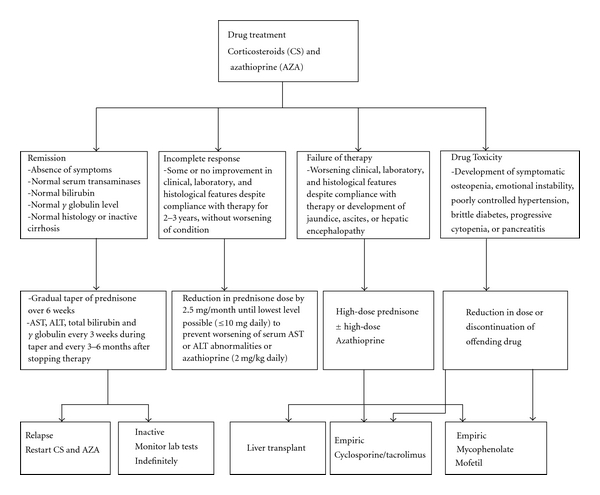
Endpoints for Immunosuppressive treatment with course of action in AIH.

Prednisone alone (60 mg daily with taper down to 20 mg daily in 4 weeks) or at a lower dose (30 mg with taper down to 10 mg daily in 4 weeks) in combination with azathioprine (50 mg daily) is the most effective treatment regimen studied in randomized clinical trials [76]. The preferred regimen is listed in Table 4. Both regimens are similarly effective and differ only in the frequency of side effects. Histologic improvement lags behind clinical and laboratory resolution by 3 to 8 months, and therapy should be continued for at least 3–6 months beyond this point of improvement. Treatment is often maintained for at least 2 years before withdrawal of drug therapy is considered. The end points for treatment include remission, treatment failure, incomplete response, or development of drug toxicity. Their criteria and subsequent intervention are summarized in Figure 2.

 Prednisone is used alone in patients with severe cytopenias, active malignancy, pregnant or contemplating pregnancy, and those with complete thiomethylpurine transferase (TPMT) enzyme deficiency. Combination therapy is associated with lesser side effects and is preferred when treatment is expected to be more than 6 months and in patients at risk of side effects including postmenopausal women, brittle diabetics, labile hypertensive, and osteoporotic patients. 

 Therapy may span over several years and hence treatment side effects must be taken into consideration. Corticosteroids can cause weight gain, central obesity, moon facies, prominent supraclavicular fat pad, acne, bruising, cutaneous striae, cataracts, glaucoma, peptic ulcers, deterioration of hypertension and diabetic control. Long-term side effects include increased risk of fractures secondary to osteoporosis and avascular necrosis of bone. Patients with brittle diabetes, severe osteoporosis, vertebral compression fractures, psychosis, obesity, and uncontrolled hypertension should be carefully evaluated for a treatment benefit before starting corticosteroids. If severity of disease necessitates corticosteroid therapy, adequate measures should be instituted to control the comorbid conditions [56]. In patients with mild disease or relative contraindications to prednisone, budesonide 3 mg TID (in place of prednisone) is an option to reduce overall treatment side effects with no impairment of efficacy [77, 78]. Its benefits are derived from the 90% first pass metabolism which results in less steroid-induced side effects while maintaining efficacy.

### 5.3. Alternative Treatments

Alternative regimens must be considered in several circumstances: after treatment failure with prednisone (60 mg daily) or prednisone (30 mg daily) and azathioprine (150 mg daily), incomplete response to conventional therapy, or intolerance to conventional therapy. Mycophenolate mofetil (2 g daily) has shown improvement in 39–84% patients who were unable to tolerate azathioprine but use was limited by side effects (nausea, vomiting, rash, pancreatitis, diarrhea, cytopenia) [79–81]. Patients in these studies were also treated with steroids in conjunction with Mycophenolate mofetil. There are studies demonstrating benefit to the use of cyclosporine (in conjunction with prednisone) for patients refractory to standard therapy. In addition, a report suggests some benefit to tacrolimus, but has not been evaluated in randomized clinical trials [82]. The risk of toxicity must be weighed with these and other agents. Ursodeoxycholic acid has been studied in randomized trials and unfortunately was not found to be of benefit [83].

### 5.4. Treatment of Relapse

Relapse is characterized by an increase in the serum aminotransferase levels to at least threefold normal. Relapse occurs in 50% to 86% of patients, most often during the first 6 months after the termination of therapy (50%). The first relapse after drug withdrawal should be retreated with a combination of prednisone plus azathioprine at the same treatment regimen as with the initial course of therapy and then tapered to monotherapy with either azathioprine (2 mg/kg daily) as a long-term maintenance therapy or indefinite low-dose prednisone (10 mg daily) in patients intolerant of azathioprine. Gradual withdrawal from maintenance therapy may be attempted again after at least 24 months of treatment and continued normal serum AST or ALT level only after careful benefit risk evaluation in patients who had previously relapsed. Repeated relapse (>2 times) has been associated with worse outcomes [84].

### 5.5. Liver Transplantation (LT)

AIH is the underlying cause for 4%–6% cases of liver transplants done in the Western world [85, 86]. It usually results from a failure to diagnose and treat AIH as an etiology of cirrhosis, inadequate response or intolerance to immunosuppressive therapy, or noncompliance with treatment. Treatment failure requiring transplant is more often associated with the HLA genotype DRB1*0301 [87]. Liver transplantation should be considered in patients with AIH and acute liver failure, decompensated cirrhosis with a MELD score ≥15, or hepatocellular carcinoma meeting criteria for transplantation [56]. LT for AIH is very successful with 10-year patient survivals of approximately 75% [88]. A combination of prednisone and a calcineurin inhibitor (tacrolimus more frequently than cyclosporine) is the most common immunosuppression regimen after LT. Despite this, AIH can recur in transplanted livers or occur de novo in liver transplants done for non-AIH conditions, but discussion of this is beyond the scope of this review article.

## 6. Other Important Dimensions of AIH

### 6.1. Pregnancy and AIH

AIH can improve during pregnancy, and this may enable reduction in immunosuppressive therapy. The greatest risk is prematurity, but fetal mortality has been reported to be as high as 21% [89]. Occurrence of adverse outcome of any type is 26%. Perinatal mortality is 4%, and maternal mortality 3% [90]. Maternal antibodies to SLA and extractable nuclear antigens (Ro/SSA) have been associated with a more complicated pregnancy [89]. Preconceptional counseling is advised and immunosuppressive therapy should be modified if possible. Azathioprine is an FDA category D drug and safety in pregnancy has not been well established in human studies. Although increased number of birth defects have not been reported in neonates of women receiving this treatment and no adverse consequences of breast feeding have been noted by treated mothers [91, 92], congenital malformations have been reported in pregnant mice, and, thus there is a potential risk for teratogenicity. This justifies exercising caution when using in pregnancy, thus the mainstay of treatment in pregnancy is prednisone at as low dose as possible. AIH commonly exacerbates following delivery, therefore therapy must be resumed (if stopped) or increased 2 weeks prior to anticipated delivery and continued in the postpartum period. 

Women with advanced cirrhosis and portal hypertension are at high risk for variceal hemorrhage during pregnancy [91]. Pregnancy should be avoided and effective contraception should be advised in these patients.

### 6.2. AIH, Cirrhosis, and Hepatocellular Carcinoma (HCC)

AIH is associated with chronic inflammation that may proceed to cirrhosis and end-stage liver disease which also puts AIH patients at risk of developing HCC. However, unlike other cohorts of cirrhotic patients, the majority of patients with AIH respond well to immunosuppression and in those whom enter a sustained remission, the potential exists to retain a near normal life expectancy. However, the interactions of disease activity, response to treatment, and other factors in relation to the risk of HCC development in AIH are unknown. Although the development of HCC in patients with AIH and cirrhosis is considered a rare occurrence, the true incidence remains unknown due to the paucity of published data addressing this issue. A large prospectively obtained cohort at a single center (*n* = 243) determined a rate of HCC development of 1.1% per year, with equal proportions among men and women [92]. The median duration from time of confirmed cirrhosis to a diagnosis of HCC was 102.5 months (range 12–195 months). Not surprisingly, HCC was found to occur more frequently in patients with cirrhosis at presentation (9.3% versus 3.4%, *P* = .048) or history of variceal bleed as the index presentation of AIH (20% versus 5.3%, *P* = .003). Median survival in patients whose HCC was diagnosed on surveillance was higher (19 months versus 2 months) compared with patients presenting symptomatically (*P* = .042) [92]. The majority of patients develop HCC after having cirrhosis for an average of 9 years, and although the incidence of HCC is less common than in other chronic liver diseases, the risk may be sufficient to undertake surveillance in all patients with cirrhosis with AIH who are candidates to undergo curative therapies.

## 7. Conclusion and Future Perspectives

Autoimmune hepatitis is one of the few liver diseases with excellent response to therapy. On the other hand, it still remains a liver disease with many unanswered questions, particularly in respect to its etiology and pathogenesis. There is significant heterogeneity in its presentation that may mask its identity, affect its clinical behavior, and confound its management. It may start with a fulminant course, and the diagnosis should not be overlooked when dealing with patients with acute liver failure. Alternatively, it may behave as a slowly progressing disease, and it is still controversial whether those patients need immunosuppressive treatment at all. There is no prescribed minimum or maximum duration of treatment. Over the last decade, remarkable progress has been made in understanding and clarifying the areas of diagnosis with introduction of classification criteria, and broadening therapeutic options, with trial of several new medications like budesonide and mycophenolate mofetil, and more in the pipeline. Management, however, still faces several other important issues, such as in children, the elderly, in males, and during the preconception period, pregnancy, and lactation. A key to successful management is thinking of it, recognizing the nonclassical presentations, and individualizing therapy.

## Figures and Tables

**Table 1:** HLA associations in autoimmune hepatitis.

HLA Association (Reference)	Ethnicity/Comments	AIH Type	Number of patients studied	Patients	Controls
HLA-DRB1*0401 [15]	(i) European and North American Increases susceptibility to AIH Type I in Caucasians (ii) HLA-DR3 associated with younger age at presentation, diminished response to therapy and more frequent liver failure requiring liver transplantation as compared to HLA-DR4	I	119	45%	23%
HLA-DRB3*0101 [15]	European and North American	I	119	58%	25%
HLA-DRB1*0404 [16]	Mexican	I	30	36.7%	7.4%
HLA-DRB1*0405 [17]	Japanese	—	49	67.3%	29.6%
HLA-DRB1*07 [18]	Brazil	II	28	68%	20%
HLA-B14 [19]	Germany	II	19	26%	4%

**Table 2:** Autoantibodies in autoimmune hepatitis.

Autoantibody	Molecular target	Prognostic value	Reference
Antiliver kidney microsomal (LKM1)	Cytochrome 450 2D6	Diagnostic for AIH type 2	[27]

Anti-LKM3	Uridine diphosphate glucuronosyltransferase	7% of patients with AIH associated with viral hepatitis C	[28]

Antismooth muscle antibody	Actin and non-actin components (vimentin, Skeltin)	Diagnostic marker for type 1 AIH	

Antiactin	Polymerized F-actin	(1) Subset of smooth muscle antibodies (2) Children: treatment dependence and progression to liver failure (3) Adults: early onset and severe disease (4) Severe clinical and histological disease if reactive to actin and *α*-actinin, anti-ss DNA antibodies can be seen as well.	[29–31]

Anti-soluble liver antigen (SLA)	Sep (O-phosphoserine) tRNA: SEC(selenocysteine) tRNA synthase	(1) High specificity, may be present when other markers are absent (2) Predictor of relapse and treatment dependence (3) Associated with DRB1*0301 (4) Higher frequency of death from liver failure.	[32, 33]

Antiliver cytosol type 1 (LC1)	Formiminotransferase cyclodeaminase	(1) Present when other markers like ANA, SMA, LKM1 absent (2) Early age of onset and concurrent immune disease (3) Marked liver inflammation and rapid progression to cirrhosis	[34, 35]

Anti-asialoglycoprotein receptor (ASGPR)	Asialoglycoprotein receptor	(1) Correlate with histological activity	[36]

Antibody to histone and double stranded DNA (dsDNA)	Histone, dsDNA	(1) Patients with anti-dsDNA fail corticosteroid treatment more frequently	[37]

Anti-chromatin	Chromatin	(1) Occur in association with ANA (2) May define a subset of ANA positive patients that are treatment dependent (3) Predictor of relapse after drug withdrawal (4) Higher levels of *γ* globulin and IgG at presentation	[38]

Perinuclear antinuclear neutrophil cytoplasmic antibodies (pANCA)	Peripheral nuclear membrane component	Seen in type 1 AIH may help in diagnosis if other tests are negative.	[39]

**Table 3:** Indications for treatment of autoimmune hepatitis.

	Absolute	Relative	None
Clinical	Incapacitating symptoms	Symptoms (Fatigue, arthralgia, Jaundice, Abdominal Pain)	Asymptomatic

Laboratory	AST ≥ 10 fold ULN AST ≥ 5 fold ULN and HG ≥ 2 fold ULN	AST or HG less than absolute criteria	Normal or near normal AST and *γ* Globulins

Histology	Bridging necrosis or Multiacinar necrosis on Histology	Interface hepatitis	Inactive cirrhosis or mild portal hepatitis

*Relative Contraindications to immunosuppressant therapy-Osteopenia, Emotional Lability, Hypertension, Diabetes, Mild Cytopenia

*Absolute Contraindications to Azathioprine or Prednisone-Vertebral compression, Psychosis, Uncontrolled hypertension, Brittle Diabetes, Severe Cytopenia (WBC count < 2.5 × 109/L, Platelet count < 50 × 109/L), Complete deficiency of Thiopurine methyl-transferase enzyme, Known intolerance to prednisone or azathioprine

AST-Aspartate aminotransferase HG-Hypergammaglobulinemia ULN-Upper limit of normal.

**Table 4:** Treatment regimen for autoimmune hepatitis.

Combination therapy		Monotherapy
Prednisone (mg/day)	Azathioprine (mg/day)	Prednisone (mg/day)
30 mg × 1 week	50 mg	60 mg × 1 week
20 mg × 1 week	50 mg	40 mg × 1 week
15 mg × 2 weeks	50 mg	30 mg × 2 weeks
10 mg maintenance dose	50 mg	20 mg maintenance dose
